# Multifactor analysis of delayed absorption of subretinal fluid after scleral buckling surgery

**DOI:** 10.1186/s12886-021-01853-2

**Published:** 2021-02-15

**Authors:** Kejun Long, Yongan Meng, Jing Chen, Jing Luo

**Affiliations:** 1 Department of Ophthalmology, The Second Xiangya Hospital, Central South University, Changsha, China; 2 Hunan Clinical Research Center of Ophthalmic Disease, Changsha, China; 3 Zhongshan Ophthalmic Center (ZOC), Sun Yat-sen University, Guangzhou, China

**Keywords:** Rhegmatogenous retinal detachment, Scleral buckling, Subretinal fluid, Choriocapillaris flow density, Optical coherence tomography, Optical coherence tomography angiography

## Abstract

**Background:**

The purpose of this study is to assess the absorption of subretinal fluid (SRF) after scleral buckling (SB) surgery for the treatment of rhegmatogenous retinal detachment (RRD). We also examined related factors that may affect the delayed absorption of SRF.

**Methods:**

This retrospective study included patients who underwent successful SB surgery for the treatment of macula-off RRD and in which the retina was reattached after the surgery. The patients were categorized according to gender, duration, age, the number, and location of retinal breaks. Subfoveal choroidal thickness (SFCT), height of subretinal fluid (SRFH), and the choriocapillaris flow density (CCFD) within 3 × 3 mm macular fovea were included. Delayed absorption was determined by the SRF that remained unabsorbed for 3 months after the procedure. The endpoint was determined when the SRF could no longer be observed.

**Results:**

A total of 62 patients (63 eyes) were enrolled. In 35 eyes (56.45%) SRF was completely absorbed and in 28 (43.55%) eyes delayed absorption of SRF in macular areas was observed at 3 months after surgery. A young age (< 35 years), inferior retinal breaks were associated with good outcomes by applying multivariable analysis on the rate of SRF absorption after SB instead of gender, the number of breaks, and duration (*p* < 0.05). CCFD was significantly different between the SRF group and the non-SRF group after SB (0.66 ± 0.04% vs 0.63 ± 0.05%, *P* < 0.05). SRFH showed a moderate positive correlation with SFCT (r_s_ = 0.462, *p* = 0.000), however, using binary logistic regression analysis it was determined that SFCT was not related to the absorption of the SRF.

**Conclusions:**

The absorption of SRF after SB may be correlated with choriocapillaris flow density. Age and location of breaks are significant factors affecting the absorption of SRF. The duration of disease is an uncertain factor due to several subjective reasons.

## Background

Scleral buckling (SB) is a traditionally effective surgery for the repair of uncomplicated retinal detachment (RD) and has a success rate of 85–95% [1]. The incidence of subretinal fluid (SRF) after SB is generally within the range of 27–78% [2–5], whereas it also yielded 94% in a specific reported case [6]. Persistent subretinal fluid (PSF) was identified by optical coherence tomography (OCT) in patients who have undergone successful surgery for RD, which cannot be seen on slit-lamp clinical examination [3, 7], and this phenomenon became more common after SB surgery than that after pars plana vitrectomy (PPV). The reason for its occurrence and development is not clear. As the PSF affects the recovery of postoperative visual function, the discussion of its related factors is helpful to provide theoretical guidance for the prevention, prediction, diagnosis, and treatment of SRF after SB surgery. The purpose of this study is to investigate the absorption of SRF after SB surgery for rhegmatogenous retinal detachment (RRD) and its affecting factors, including choroidal thickness. Choriocapillaris flow density, age, gender, duration, the number, and position of retinal breaks.

## Methods

This retrospective observational case series was approved by the Institutional Medical Ethics Board of the Second Xiangya Hospital of Central South University and adhered to the tenets of the Declaration of Helsinki. The informed consent of the patient was exempted by the Institutional Medical Ethics Board of the Second Xiangya Hospital of Central South University. All data were recorded and stored in compliance with ethical and data protection guidelines.

In this study, the medical records of patients with primary RRD involving the fovea (macula-off) were reviewed. These patients were treated with SB surgery (with retina reattachment after surgery) between March 2016 and October 2019 at the Second Xiangya Hospital of Central South University.

Inclusion criteria included: primary RRD; macula-off RRD confirmed by OCT; underwent SB surgery (including radial/circumferential buckle, segmental circumferential or encircling); no other treatments, such as retinal photocoagulation, intravitreal injections, or intraocular gas filling performed during the follow-up period. Exclusion criteria included: macula-on RRD; nonrhegmatogenous retinal detachment; previous or present underlying macular disease or retinal vascular disease; refractive media opacity that could not be examined during the follow-up period; a history of previous intraocular surgery (except for simple cataract extraction); or a history of ocular trauma.

All patients were treated with standard SB surgery. Before surgery, the pupil was dilated and the retinal breaks were examined by an indirect ophthalmoscope and a three-mirror contact lens.

The surgical steps are shown as follows: cut the conjunctiva 2–3 mm posterior to the limbus. Open the sub-Tenon’s space to expose the bare posterior sclera and isolate the extraocular muscles with a muscle hook. Between two and four rectus muscles are slung depending on the location of breaks and the planned size of the buckle which we checked preoperatively. Traction sutures with 10–0 black silk are placed underneath each muscle. The fundus examination with a 20D indirect ophthalmoscope is done to identify the location of all retinal breaks and additional vitreoretinopathy. Once identified, all retinal breaks are treated with cryopexy. After the application of cryotherapy to retinal break(s), a radially or circumferentially oriented scleral explant (silicone sponge/solid silicone tires) is positioned to seal the break. A radial or circumferential sponge is used to close a single horseshoe. The circumferential segment is preferably used in cases with multiple breaks and retinal dialysis. Encircling is preferably used in cases with 360° lattices and atrophic holes. According to the principle of minimum surgical volume, buckling material was sutured to the sclera with a mattress suture using 5–0 ethibond.

All patients were followed up after 7 days, 1, 3, 6, and 12 months with OCT and Angio optical coherence tomography (OCTA). The disappearance of PSF was considered as the follow-up endpoint. In this study, the definition of absorption is that the subretinal fluid is completely absorbed 3 months after the surgery, and delayed absorption means that the subretinal fluid has not been absorbed 3 months after the surgery. The presence of subretinal fluid means that subretinal fluid is visible at each follow-up, the absence of subretinal fluid means that no subretinal fluid is observed at each follow-up.

**Fig. 1:**
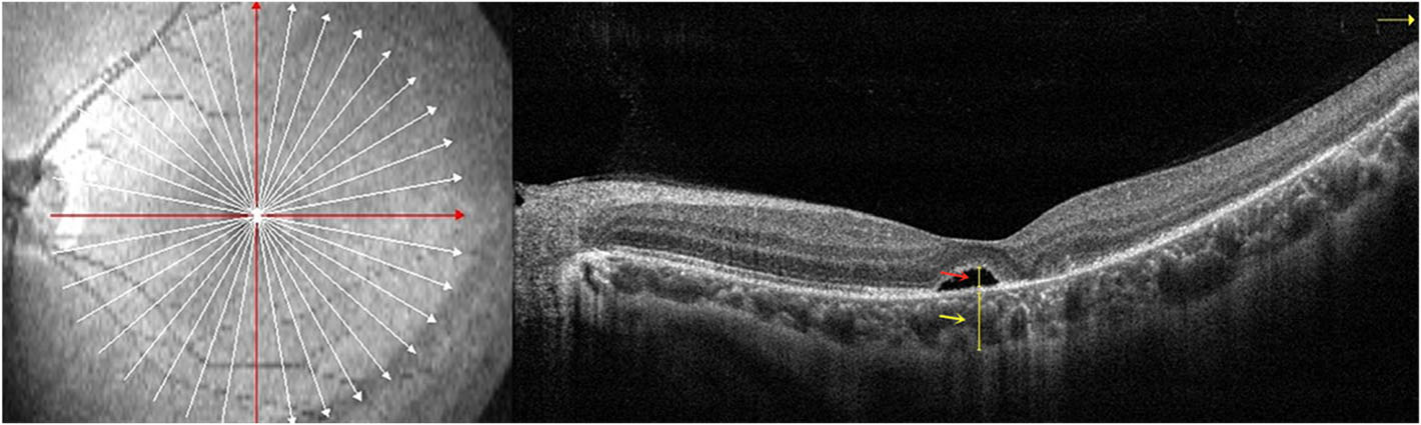
Measurement of subfoveal choroidal thickness and the height of subretinal fluid in the macular fovea SFCT represents the vertical distance from the outer surface of the foveal pigment epithelium to the inner surface of the sclera (yellow arrow). SRFH corresponds to the vertical distance from the outer interface of the neuroepithelial layer to the inner surface of the pigment epithelium (red arrow)

All scans were performed by experienced OCT operators. The retinal macular area was scanned using the Radial Line Mode in OPTOVUE OCT. Using this method, we could obtain the horizontal and vertical images that spanning the macular fovea, then performed measurements. The subfoveal choroidal thickness (SFCT) was manually measured using the built-in ruler in the software. This measurement was assessed by determining the vertical distance from the outer surface of the foveal pigment epithelium to the inner surface of the sclera (as indicated by the yellow arrow in Fig. 1). SRFH under the macular fovea was also manually measured by determining the vertical distance from the outer interface of the neuroepithelial layer to the inner surface of the pigment epithelium (as indicated by the red arrow in Fig. 1). The dividing line was assessed independently by three different physicians and the measurement reported is the average value of the three measurements. If there was a significant difference between the measurements, the data were excluded. This measurement protocol was repeated at each follow-up examination.

**Fig. 2:**
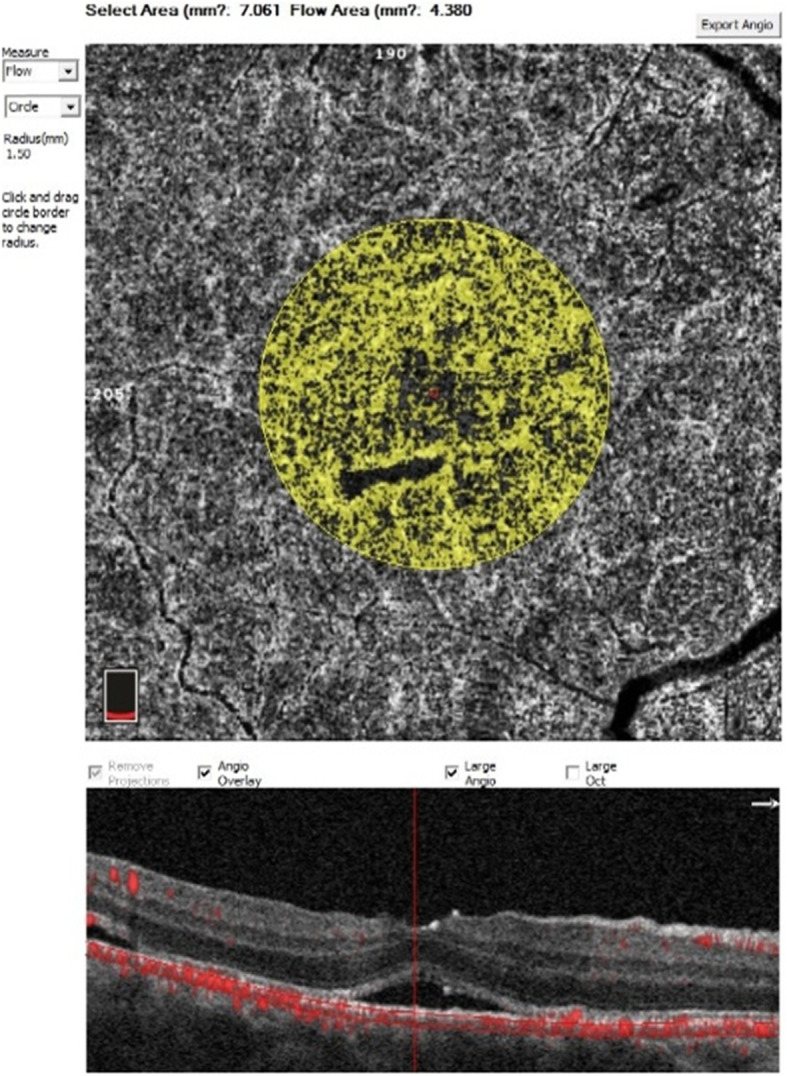
Measurement of choriocapillaris flow density Using the OPTOVUE HD Angio Retina mode the blood flow area was measured in the circular range of 1.50 mm radius of the choriocapillaris layer. CCFD was determined by dividing the blood flow area by the selected area

The retinal macular area was scanned with OPTOVUE HD Angio Retina mode. The area of choriocapillaris blood flow (CCFD) was obtained with a scanned image of a 3 × 3 mm range within the macular fovea. Acquired CCFD was determined by dividing the blood flow area by selected area (as shown in Fig. 2).

All the patients were divided into group A (male) and group B (female) according to gender. Patients were further grouped: group C (duration ≤1 week (w)) and group D (duration >1w) based on the duration of disease; group E (< 35 years) and Group F (≥ 35 years old) according to age; group G (1 break) and group H (multiple breaks) based on the number of retinal breaks; and the group I (inferior, retinal break that located between 3 and 9 of clock-hours in the retina) and J (non-inferior) based on the location of the retinal breaks. Comparisons were made regarding absorption of SRF between each set of groups at 3 months after surgery.

The statistical analyses were performed with the IBM SPSS23.0 (Version 23; SPSS Inc., Chicago, IL, USA) package. The measurement data were represented as mean ± standard deviation. The correlation between SRFH and SFCT as well as SRFH and CCFD was analyzed by Spearman’s correlation analysis. SFCT and CCFD data were divided into SRF groups and non-SRF groups, then compared these two groups by two independent samples t-test. The effect of SFCT and CCFD on the absorption of SRF was analyzed by binary logistic regression (α_in_ = 0.05, α_out_ = 0.10). The absorption of SRF in a different age, gender, duration, number, and position of retinal breaks were determined by Fisher’s exact test. The influence of the above five factors on the absorption of SRF was analyzed by binary logistic regression (α_in_ = 0.05, α_out_ = 0.10).

## Results

A total of 63 eyes of 62 patients were included in this study (Table 1). All patients were successfully treated with SB surgery and the retina was recovered after the surgery. The age of the patients ranged from 12 to 70 years old, with an average age of 40.06 ± 17.132 years. Regarding gender, 30 eyes (48.39%) were from male patients and 32 eyes (51.62%) were from female patients. When divided according to age, 25 eyes (40.32%) were from patients under 35 years old, and 37 eyes (59.68%) were from patients older than 35 years old. Regarding the duration of the disease course, 23 eyes (37.10%) had a disease less than 1 week and 39 (62.90%) had a disease for more than 1 week. When divided by retinal breaks, 47 eyes (78.33%) had a single break and 13 eyes (21.67%) had more than one break.

**Table 1:** General data of patients (percentage accurate to 2 decimal places)

	n(%)
Gender	
Male	30 (48.39)
Female	32 (51.62)
Age	
<35y	25 (40.32)
≥ 35y	37 (59.68)
Diseases duration (time of RD)	
≤ 1w	23 (37.10)
>1w	39 (62.90)
Numbers of retinal breaks	
1	47 (78.33)
>1	13 (21.67)
Location of retinal breaks	
Inferior	15 (24.19)
Non-inferior	47 (75.81)
Absorption time of SRF	
≤ 3 m	35 (56.45)
>3 m	28 (43.55)

When divided according to the location of the retinal break, 15 eyes (24.19%) had inferior breaks and 47 eyes (75.81%) had broken in non-inferior regions. Lastly, regarding the absorption time of SRF, 35 eyes (56.45%) had a decrease in subretinal fluid absorption for less than 3 months and 28 eyes (43.55%) had a decrease for more than 3 months.

**Fig. 3:**
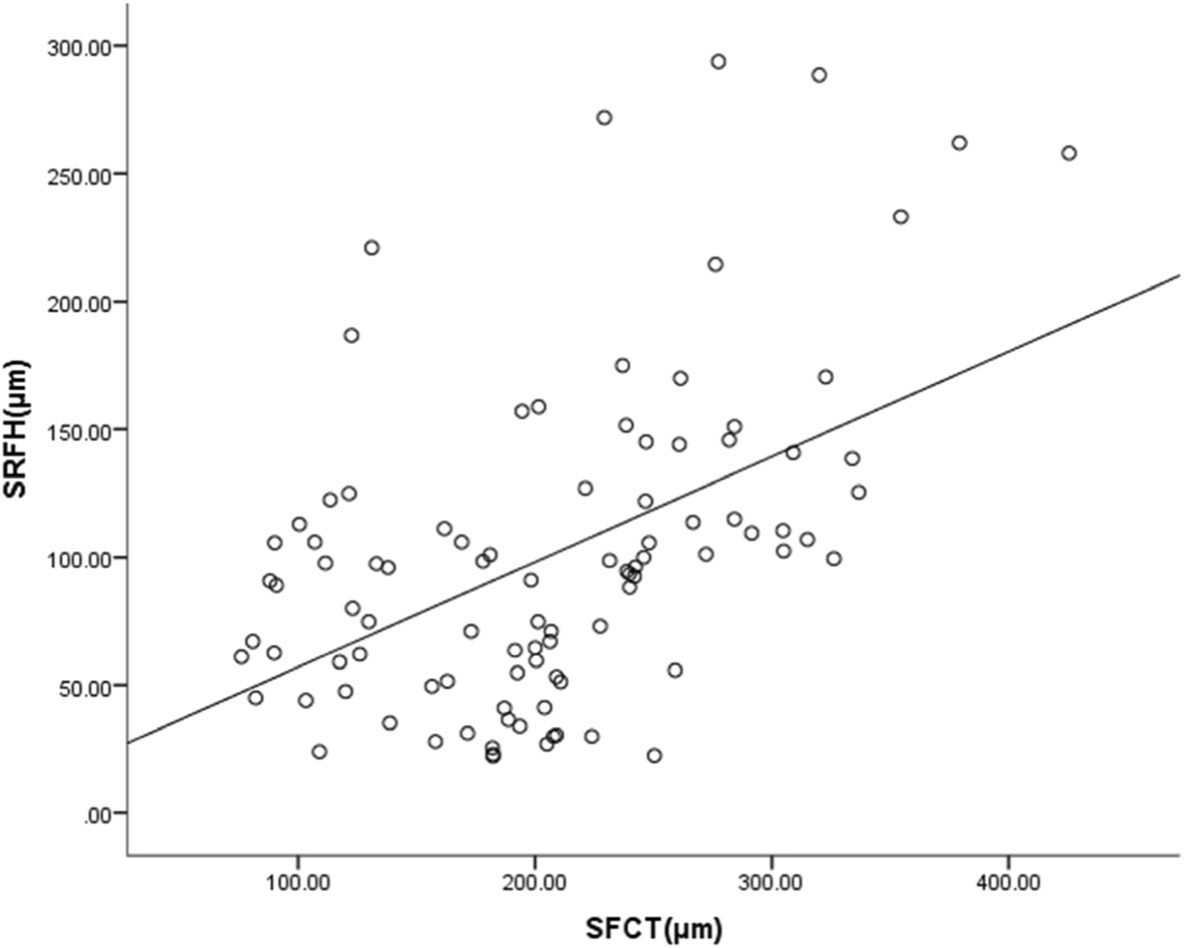
Scatter diagram of a correlation between SRFH and SFCT in the macular fovea, where SRFH shows a moderate positive correlation with SFCT (rs = 0.462, *p* = 0.000)

There was a moderate positive correlation between SFCT and SRFH (r_s_ = 0.462, *p* = 0.000, Fig. 3).

There was no significant correlation between CCFD and SFCT (*P* > 0.05). SFCT and CCFD conformed to a normal distribution, and there was a significant difference in CCFD between the non-SRF group and SRF group (0.66 ± 0.04% vs 0.63 ± 0.05%, *p* < 0.05). However, there was no significant difference in SFCT (*p* > 0.05). Binary logistic regression analysis (non-SRF was 1 and SRF was 0) showed that CCFD was a significant factor affecting the absorption of SRF (OR = 1.123; 95% CI 1.002, 1.259; *p* = 0.046) (Table 2).

**Table 2:** Logistics regression analysis of the effects of SFCT and CCFD on SRF absorption

	β	Error	Waldχ^2^	Sig	OR	95% CI	
						Lower-bound	Upper-bound
SFCT	−.001	.003	.100	.752	.999	.993	1.005
CCFD	.116	.058	3.970	.046	1.123	1.002	1.259

SRF absorption after SB was statistically different when comparing the groups according to age and retinal break location (*p* < 0.05, Table 3). While the groups of gender, duration of disease, and the number of breaks exhibited less association with the SRF absorption (*p* > 0.05). Difference refers to comparison within groups rather than comparison between groups. Binary logistic regression analysis showed that age and location of retinal breaks were significant factors that affect the absorption of SRF. The incidence of delayed SRF absorption in the younger age (< 35) group was 4.56 times higher than in the older age (≥ 35) group (OR = 4.555; 95%CI 1.388, 14.946; *p* = 0.012). Likewise, the incidence of delayed absorption of SRF in the inferior break group was 3.40 times higher than that in the non-inferior break group (OR = 3.398; 95%CI 0.835, 13.829; *p* = 0.088) (Tables 4 and 5).

**Table 3:** Influence of various factors on the absorption of SRF

	Absorbed (n)	Unabsorbed (n)	Rate of absorption (%)	X^2^	P
Age				11.015	0.002
<35y	8	18	31		
≥ 35y	27	10	73		
Gender				0.029	1.000
Male	17	13	57		
Female	18	15	55		
Duration				0.272	0.791
≤ 1w	12	11	52		
>1w	23	16	60		
Numbers of retinal breaks				0.054	1.000
1	27	20	57		
>1	7	6	44		
Location of retinal breaks				7.141	0.015
Inferior	4	11	27		
Non-inferior	31	16	66

**Table 4:** Five risk factors and assignment affecting SRF absorption

Factor	Variable name	Assignment
Age	X1	<35 = 1, ≥ 35 = 0
Location of retinal breaks	X2	inferior = 1, non-inferior = 0
Duration	X3	>1w = 1, ≤ 1w = 0
Number of retinal inferior	X4	>1 = 1, 1 = 0
Gender	X5	female = 1, male = 0
Delayed absorption of SRF	Y	yes = 1, no = 0

**Table 5:** Logistic regression analysis of the effects of five factors on SRF absorption

	β	Standard error	Wald χ2	Sig	OR	95%CI	
						Lower-bound	Upper-bound
X1	1.516	.606	6.256	.012	4.555	1.388	14.946
X2	1.223	.716	2.918	.088	3.398	.835	13.829
X3	−.870	.713	1.487	.223	.419	.103	1.696
X4	−.211	.746	.080	.777	.809	.187	3.495
X5	1.114	.737	2.286	.131	3.047	.719	12.920

## Discussion

RRD, which is the detachment of the retinal neuroepithelial layer from the retinal pigment epithelium (RPE) caused by breaks in the retina. This allows fluid from the vitreous to enter the cavity of subretinal, which can cause permanent loss of vision if left untreated [8, 9]. Previous studies have reported poor visual recovery and high incidence of persistent subretinal fluid (PSF) in patients with macula-off RRD [10]. In this study, SRF was completely absorbed in 35 eyes (56.45%) within 3 months after the operation, and 28 eyes (43.55%) had PSF in the macular fovea. These patients commonly had PSF after scleral compression. The mechanism of PSF has only been minimally examined in previous studies. Possible mechanisms have been proposed include the redundant retina after SB, the dysfunction of retinal pigment epithelium (RPE) pumping function, the reduction of blood flow in choroid due to the buckle, and the difficult nature of absorption of postoperative residual liquid with a high viscosity under the retina by the RPE [11–14]. Other studies have suggested that the incidence of PSF is related to the selection of scleral buckle or segmental scleral buckle, the use of intraocular tamponade, the complete drainage of SRF, and the use of cryotherapy during surgery. However, the above conclusions were still under debate [15, 16].

In this study, the effects of age, gender, duration of disease, number, and position of breaks on the absorption of SRF were analyzed. The results suggest that gender, the number of breaks, and the duration of the disease show fewer differences in the absorption of SRF. And some studies have shown that the duration of disease does not affect the absorption of SRF [17–19]. However, we must take into account that the deviation in some patients, especially those with inferior breaks, maybe due to the subjective judgment of the assessment of the course of the disease or complicated due to the symptoms that are not obvious.

Binary logistic regression analysis showed that only age and the position of breaks were significant factors affecting the absorption of SRF. The delayed absorption probability of SRF in the inferior group was 3.40 times higher than that in the non-inferior group. This is consistent with Veckeneer’s view that the inferior RD is more prone to postoperative PSF, and that the duration is longer [15, 20, 21]. Prolonged retentions of vitreous diffusion into the subretinal fluid may lead to the re-modification of protein components and macromolecules [22, 23], resulting in decreased absorption of SRF by RPE. When the retina is detached for a significant time a cystic space is formed in the retina and the photoreceptors degenerate and the retina atrophies. This hinders the absorption of SRF. In our study, the absorption of SRF after SB was compared in groups of patients with inferior retinal breaks and the non-inferior retinal breaks (*P* < 0.05). When breaks were located in the inferior retina, the incidence of PSF was higher, and the incidence of delayed absorption of SRF in the group with inferior breaks was 3.40 times greater than the group with non-inferior breaks.

Park et al. proposed that the 35 years of age was the inflection point of bimodal RRD incidence [24]. Therefore, in our study, patients were sorted into two groups by age (< 35 and ≥ 35). Our results showed that the absorption of SRF after SB was statistically significant when compared with the older (≥35) and younger age (< 35) group (*P* < 0.05). The incidence of SRF absorption in the older age (≥35) group was 4.56 times of that in the younger age (< 35) group, suggesting that the incidence of delayed SRF absorption in younger patients was higher. We speculate that younger patients have less vitreous liquefaction and higher viscosity, which makes the absorption of SRF in the potential subretinal space become more difficult [25]. Furthermore, younger patients may have less liquefied and more viscous vitreous, which can block retinal breaks, making the PSF more likely to occur and more difficult to absorb [26]. The incidence of PSF after PPV is less than that of SB, which also supports the hypothesis that the vitreous state may be a factor affecting SRF absorption [27]. Hyaluronic acid is an important component of the vitreous. SRF contains a significant amount of hyaluronic acid, which may slow the absorption of liquid by inhibiting the phagocytosis of RPE [28]. As age increases, the level of hyaluronic acid in the vitreous decreases, possibly reducing the incidence of PSF. Some studies have also proposed that the long-term persistence of SRF may lead to changes in proteins and other components, and reduce the absorption rate of SRF by RPE. Proteases in SRF make cause RPE cells migrate to SRF, breaking the integrity of RPE and blood-retinal barrier, and increasing vascular permeability. Thus, this may delay the absorption of subretinal fluid [29]. Moreover, RRD in young patients tends to have shallow detachment and was reported to show a correlation with better preoperative visual acuity. Less visual loss and slow progression could be related to late recognition and longer symptom duration [26]. Veckeneer M et al. speculated that younger age was not an independent factor affecting the delayed absorption of SRF. Sustained SRF was more frequent in the young patient, which could be related to long symptom duration [15]. However, previous study young patients with superior retinal detachments also have postoperative subfoveal fluid on OCT at 1 month postoperatively.

With the application of OCT and OCTA, this study was able to explore the effects of choroidal thickness and choriocapillaris blood flow density (CCFD) on SRF. Although the fundus variation caused by RRD and the surgery are mainly in the retina, some studies have reported that the choroidal structure also changed. Odrobina D et al. observed changes in the choroid after SB surgery by EDI-OCT, and report that the choroid in the central fovea was significantly thickened after the operation. It is worth considering that the width and size of materials used during the operation may cause choroidal blood flow stasis, reduce choroidal flow, and lead to the choroidal vascular pressure increase, thereby increasing the thickness of subfoveal choroid [30, 31]. In a study of central serous chorioretinopathy (CSC), Lee J et al. proposed that the pathogenesis of SRF may be due to the high permeability of choroidal vessels, as well as the increase of colloidal osmotic pressure caused by choroidal thickening [32]. Kim J et al. reported a choroidal hyperosmotic condition after the repair of RRD, and the choroidal thickness was thicker than that without the hyperosmolarity by indocyanine green angiography (ICGA) [12]. Chan et al. found that images with high signal density in CSC were accompanied by the dilatation of large blood vessels, suggesting that the choroidal thickening in CSC may be due to the expansion of blood vessels [33]. Therefore, it was suggested [34, 35] that the mechanism of SR after SB surgery may be similar to the pathogenesis of CSC, such as choroidal hypertonicity and choroidal thickening. The results of our study show a moderate positive correlation between SRFH and SFCT at the macular fovea (r_s_ = 0.462, *P* = 0.000). Thus, a thicker choroid leads to a higher height of SRF in the macular fovea. This suggests that choroidal thickening may delay the absorption of SRF. Regarding the factor of gender, a previous study proposed that males have a thicker choroid in comparison with females [36]. The thicker choroid in men indicates that the choroidal perfusion pressure is higher than in women. However, in the bivariate regression analysis, a thicker choroid did not affect the absorption of SRF. Similarly, Kim proposed that the choroidal thickness and number of breaks were not correlated with the absorption of SRF, who also found that the choroidal thickness shows a tendency to reduce over time. While the underlying mechanism remains unclear [12]. The CCFD was a significant factor affecting the absorption of SRF. The results of our study showed that there was a statistical difference in the CCFD average between the SRF group and the non-SRF group. The CCFD in the non-SRF group was significantly increased compared to the SRF group. Binary regression analysis suggests that for every 1% increase in CCFD a 12% increase in the incidence of SRF absorption occurs. Therefore, we hypothesis that a decrease of choriocapillaris blood flow density may lead to delayed absorption of subretinal fluid.

In this retrospective study, there are several limitations. First, in terms of experimental design, the follow-up data of some patients was missing. Second, the data distribution of each group is uneven, which may lead to unattended deviation in the results. Third, in terms of measurement, the manual measurement of choroidal thickness and height of SRF, there is a large deviation in the data.

## Conclusions

The absorption of SRF after SB may be related to the choriocapillaris flow density. The result of binary logistic regression analysis showed that that age and the position of breaks are significant factors affecting the absorption of SRF. While choroidal thickness, gender, and the number of breaks were not related to the delay of SRF absorption. The duration of the disease is often difficult to assess due to subjective reasons.

## Data Availability

All data generated or analyzed to support the findings of this study are available in the paper without restriction. The raw data during this study are available from the corresponding author on reasonable request.

## Abbreviations

SRF: Subretinal fluid
SB: Scleral buckling
RRD: Rhegmatogenous retinal detachment
SFCT: Subfoveal choroidal thickness
SRFH: Height of Subretinal fluid
CCFD: Choriocapillaris flow density
RD: Retinal detachment
PSF: Persistent subretinal fluid
OCT: Optical coherence tomography
OCTA: Angio optical coherence tomography
PPV: Pars plana vitrectomy
RPE: Retinal pigment epithelium
CSC: Central serous chorioretinopathy
ICGA: Indocyanine green angiography
